# HER2 and uPAR cooperativity contribute to metastatic phenotype of HER2-positive breast cancer

**DOI:** 10.18632/oncoscience.146

**Published:** 2015-03-23

**Authors:** Vineesh Indira Chandran, Serenella Eppenberger-Castori, Thejaswini Venkatesh, Kara Lea Vine, Marie Ranson

**Affiliations:** ^1^ Department of Clinical Sciences, Section of Oncology and Pathology, Lund University, Lund, Sweden; ^2^ Institute for Pathology, Department of Molecular Pathology, Schoenbeinstrasse, Basel, Switzerland; ^3^ Nitte University Centre for Science Education and Research (NUCSER), K. S. Hegde Medical Academy, Nitte University, Deralakatte, Mangalore, Karnataka, India; ^4^ School of Biological Sciences, University of Wollongong, Wollongong, NSW, Australia; ^5^ Centre for Medical & Molecular Bioscience, University of Wollongong, Wollongong, NSW, Australia; ^6^ Illawarra Health and Medical Research Institute, University of Wollongong, Wollongong, NSW, Australia

**Keywords:** HER2/ERBB2, uPAR/PLAUR, HER2-positive breast cancer, co-overexpression, co-amplification, correlation

## Abstract

Human epidermal growth factor receptor type 2 (HER2)-positive breast carcinoma is highly aggressive and mostly metastatic in nature though curable/manageable in part by molecular targeted therapy. Recent evidence suggests a subtype of cells within HER2-positive breast tumors that concomitantly expresses the urokinase plasminogen activator receptor (uPAR) with inherent stem cell/mesenchymal-like properties promoting tumor cell motility and a metastatic phenotype. This HER-positive/uPAR-positive subtype may be partially responsible for the failure of HER2-targeted treatment strategies. Herein we discuss and substantiate the cumulative preclinical and clinical evidence on HER2-uPAR cooperativity in terms of gene co-amplification and/or mRNA/protein co-overexpression. We then propose a regulatory signaling model that we hypothesize to maintain upregulation and cooperativity between HER2 and uPAR in aggressive breast cancer. An improved understanding of the HER2/uPAR interaction in breast cancer will provide critical biomolecular information that may help better predict disease course and response to therapy.

## INTRODUCTION

Breast cancer (BC) is a highly heterogeneous disease consisting of several subtypes, each classified by their unique biological signature [1, 2]. Each BC subtype exhibits varied responses to different therapeutic regimens. Treatment options for metastatic disease remains limited despite the availability of several United States Food and Drug Administration (FDA) approved drugs against BC [3]. In this scenario, it is imperative to explore different therapeutic models of targeting one or more tumor-specific biomarkers that define the more aggressive breast carcinoma subtypes efficiently for improved management of the disease.

Established BC biomarkers predicting metastatic risk include lymph-node involvement, hormone independency, loss of histopathological differentiation of primary tumor (grade), elevated proliferation, and angiogenesis. However, these biomarkers confidently predict outcome for only ~30% of patients. Of the remaining patients some will still develop metastases whilst others will not [4]. Components of the urokinase plasminogen activation system, particularly urokinase plasminogen activator (uPA, Gene symbol: *PLAU;* located on chromosome 10q22.2), its receptor uPAR (Gene symbol: *PLAUR;* located on chromosome 19q13) and inhibitor plasminogen activator inhibitor type 1 (PAI-1, Gene symbol: *SERPINE1;* located on chromosome 7q22.1) are proven to be associated with aggressive carcinoma. The combination of uPA/PAI-1 at the protein level is a strong and independent predictor of metastasis in lymph-node negative BC patients and predicts response to hormone therapy [5, 6]. uPAR is expressed in malignant cells and in the tumor stroma which translates into an aggressive tumor phenotype and poor relapse-free survival (RFS) [7].

The recognition of human epidermal growth factor receptor type 2 (HER2, Gene Symbol *HER2;* located on chromosome 17q12) over-expression as a therapeutic target for advanced breast carcinoma was primarily related to the clinical finding that *HER2/neu* proto-oncogene is amplified in 15–25% of all breast tumors, and is often associated with poor disease-free survival (DFS) [8-15]. The mechanism by which HER2 overexpression imparts increased aggressiveness to tumors has been attributed mostly to dysregulated activation of downstream intracellular signaling pathways [16-25]. In some cases HER2 overexpression has been reported to induce resistance to certain chemotherapeutics [26-28]. Furthermore, HER2 overexpression has been found in both in the primary tumor, circulating tumor cells (CTCs) and corresponding metastases [29-31].

A high level of correlation was observed between *HER2* and *uPAR* mRNA in disseminated tumor cells (DTCs) in 8 out of 16 patients (50%) and was associated with a more aggressive primary tumor phenotype (estrogen receptor (ER)-negative, progesterone receptor (PR)-negative or HER2-positive) [32]. Also a positive association between *HER2* and *PLAUR* gene amplification (which was concordant with protein expression in both cases) was found in >90% of HER2-amplified individual tumor cells from the blood or tissue of patients with advanced recurrent BC [33]. These and other studies [34-38] suggested the possibility of cooperativity between the HER2 and uPAR signaling pathways leading to recurrence/metastases; however the exact mechanism remains to be elucidated. Furthermore, nuclear factor-kappaB (NF-κB) mediated expression of HER2 and uPAR in cancer stem cells (CSCs), has been implicated for maintaining malignancy at the invasive edge of BC, which suggests an enhanced role for HER2-uPAR cooperative overexpression in disease relapse with an aggressive intent [39].

This review analyzes and substantiates the cooperativity between *HER2* and *PLAUR* in terms of their correlation status at the mRNA level in primary tumors of BC patients. For the first time, we also propose a regulatory signaling model as a mechanism responsible for maintaining the aggressive properties of primary and DTCs, through high co-expression of HER2 and uPA receptors and use it as a rationale to highlight the importance of simultaneously targeting HER2 and uPAR in advanced BC.

### HER2-positive BC

A working model for BC molecular taxonomy utilizing microarray-based gene expression profiling classifies BCs by hierarchical cluster analysis, using an intrinsic gene list, into four main molecular subtypes: luminal A, luminal B, basal-like, and HER2 [40-45], with subgroups increasingly being identified such as claudin-low and normal breast-like [46-49]. Each subtype displays unique patterns of metastatic spread associated with notable differences in survival after relapse [50]. Clinically, HER2-positive tumors comprise approximately 12–30% of all invasive BCs and are most often found in younger patients and associated with poorer clinical outcomes [51, 52]. This subtype is associated with increased cell proliferation, angiogenesis, tumor invasiveness, and a high nuclear grade [53]. It has been observed that patients with HER2-positive tumors are more likely to have multifocal/multicentric cancers and nodal involvement [54]. At the molecular level, HER2-positive BCs exhibits extensive changes in the patterns of gene expression associated with the HER2 pathway and/or HER2 amplicon located in the 17q12 chromosome. The manifestation of the variation in the expression of specific subsets of genes exclusive to HER2-positive BC is reflected mainly in the variation in growth rate, activity of specific signaling pathways, and in the cellular composition of the tumors [40]. Several signaling pathways are triggered in HER2-positive BC [55-57]. A detailed description of HER2-positive BC subtype can be found in Eroles et al. [49].

### uPAR expression in BC

The urokinase receptor (uPAR) is linked to the plasma membrane via a glycosyl phosphatidylinositol (GPI) anchor, which is hypothesized to enable high intramembrane mobility [58]. Upon binding uPA with high affinity (1 nM) and selectivity, co-localized zymogen plasminogen is converted to the serine proteinase plasmin thereby facilitating cell migration by tissue remodeling. uPAR interacts with other molecules disparate from its function as a proteinase receptor, including vitronectin, members of the integrin adhesion receptor superfamily, caveolin, and G-protein-coupled receptor (GPCR). As a result, uPAR activates intracellular signaling molecules such as tyrosine- and serine-protein kinases (such as EGF receptor, lymphocyte protein tyrosine kinase (Lck), haematopoietic cell kinase (Hck), Src, focal adhesion kinase (FAK) and extracellular-signal-regulated kinase (ERK)/mitogen-activated protein kinase (MAPK)) ultimately affecting migration, adhesion, differentiation and proliferation through intracellular signaling [59, 60]. Numerous clinical studies have implicated uPAR expression with phenotypically aggressive BC [61, 62] and low DFS [63]. Tumor cells (e.g. MCF-7, LNCaP) that do not express uPAR or express only low levels of uPAR were poorly tumorigenic in mice [64]. uPAR expression has often been found restricted to cells at the invasive edge of a tumor or in tumor cells at the tumor-stromal interface [65] and the expression of uPAR appears to increase with grade or stage of the tumor and may be enriched in metastatic lesions [66]. Various studies have also found uPAR to be highly expressed in CTCs from patients with advanced breast cancers [33, 38]. In addition, uPAR expression has been described in CSCs in BC [67]. For example, Jo et al., [67] showed that MCF-7 and MDA-MB-468 BC cells acquire CSC-like properties when uPAR is overexpressed and uPAR-dependent signaling is activated.

### HER2 and uPAR cooperativity in HER2-positive BC

#### Cooperation of HER2 and uPAR at mRNA level

Cooperativity between HER2 and uPAR has emerged as a strong determinant for the aggressive properties of HER2-positive BC [33, 34, 37]. Although HER2 and uPAR were described as independent tumor-specific protein predictors of BC progression for decades, correlative expression of HER2 and uPAR was first reported by Pierga et al., [32] who found a high level of correlation between *HER2* and *uPAR* mRNA in disseminated tumor cells (DTC) in 8 out of 16 patients (50%) and was associated with a more aggressive primary tumor phenotype (estrogen receptor (ER)-negative, progesterone receptor (PR)-negative or HER2-positive). Following this, Meng et al., [33] found *PLAUR* to be co-amplified with *HER2* in individual tumor cells in the blood and tissue of advanced recurrent primary BC patients. They found that if the advanced BC patients had higher *HER2* gene amplification in tumor cells from their primary breast carcinomas, then they were more likely to have co-amplification and higher levels of *PLAUR* amplification. They observed 92% (23 of 25) *PLAUR* gene amplification in *HER2* amplified cases in touch preps of primary tumor and CTCs; whereas in *HER2* nonamplified tumors, only 3% (1 of 39) were *PLAUR* gene amplified, highlighting the correlation of *HER2* and *PLAUR* gene status.

Similarly, another independent study published by Urban et al., [35], in the same year, showed that patients with *HER2*-positive/*PLAU*–positive tumors (as assessed at the mRNA level) exhibited significantly reduced metastases-free survival (MFS) compared to patients with *HER2*-positive/*PLAU*–negative tumors. This study strongly implicated uPA expression, using three independent study populations assayed by different gene expression techniques, as a powerful prognostic indicator associated with distant MFS in patients with HER2-positive tumors. This was later confirmed by Staaf et al., [36] who found *PLAU* gene status within the HER2-derived prognostic predictor (HDPP) gene signature strongly associated with basal-like, ER-negative, lymph-node positive, high grade BC. They found a significant correlation between increased mRNA and protein levels of the *PLAU* gene in tumors classified as poor by HDPP and in the data set obtained from the Nederlands Kanker Instituut (NKI), HDPP retained strong prognostic value when stratified for *PLAU* status for both overall survival (OS) and distant metastasis-free survival (DMFS). Very recently, Berg et al., [37] found significant correlation between HER2 and uPAR while analyzing protein networks in 106 formalin-fixed and paraffin-embedded (FFPE) BC tissues by reverse phase protein microarray (RPPA) analysis. Markiewicz et al., [38] found that CTC-enriched *HER2*-positive (mRNA) blood samples from lymph node positive BC patients were 100% positive for *PLAUR* mRNA expression compared to 34% of HER2 negative samples. Further, in the hierarchical clustering of the clinicopathological data, where the study population was divided into two main groups that differed in the expression of *VIM, CXCR4, PLAUR, HER2,* they found that *p*atients in the cluster with elevated expression of these genes showed more frequent lymph node involvement (58%) than patients from the cluster with lower expression (35%). All this evidence point towards a potential strong cooperativity between HER2 and uPAR resulting in enhanced metastatic potential, giving early indications of a potential synergistic co-expression.

**Figure 1:**
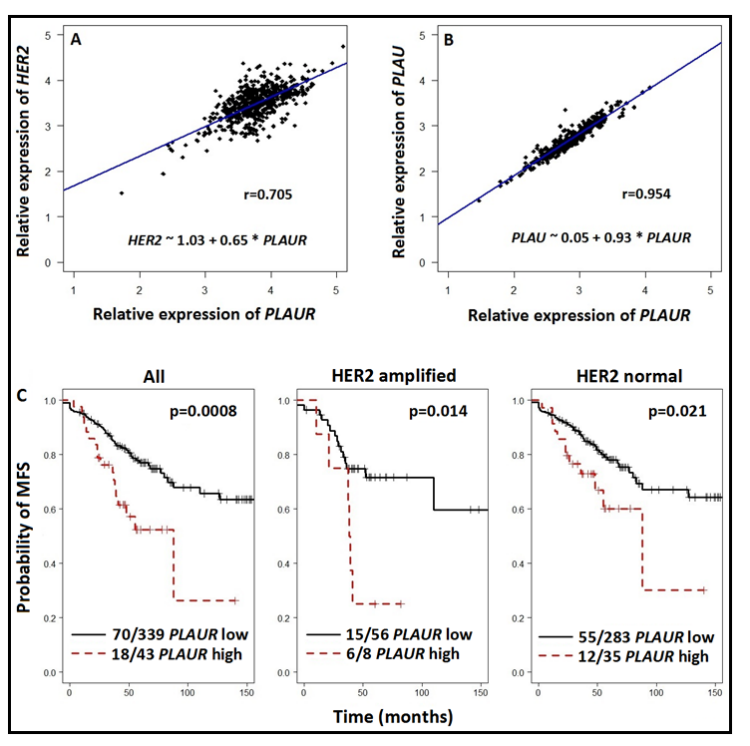
Scatter plot depicting the correlation of the relative RNA expression levels of *HER2* (A) and *PLAU* (B) versus *PLAUR*, respectively (C) Kaplan-Meier curves with respect to metastases-free survival (MFS) stratified based on low and very high *PLAUR* RNA expression levels in the overall collective, in the HER2 amplified and HER2 normal subset of patients. The curves were compared with the log-rank test and statistical analyses were performed with R (Version 2.15.2).

In order to ascertain the interdependence between *HER2* and *PLAUR* mRNA status in HER2-positive breast carcinoma, we constructed a correlation curve utilizing the *HER2* and *PLAUR* mRNA values obtained from fresh frozen tissue of the former “Stiftung Tumorbank Basel” biobank (now part of the Biobank Pathology at the University Hospital of Basel, BPUB). In this subset of 450 primary BC patients, we found the RNA expression levels of *HER2* and *PLAUR* to be strongly and significantly (r=0.705, p<0.0001) correlated (Figure 1A). An extremely strong correlation (r= 0.954, p<0.0001) between the gene expression levels of *PLAU* and its receptor *PLAUR* was also observed (Figure 1B). Patients and tumor characteristics are displayed in Table 1.

**Table 1:** Patient and tumor characteristics

Characteristics		HER2 normal		HER2 amplified	
		N=369	(82 %)	N=81	(18 %)
Age	Years: mean (range)	60	28–91	57	27–87
Histologic subtype	Invasive ductal	246	66.7%	63	70.8%
	Invasive lobular	51	13.8%	5	5.6%
	Other (mixed)	72	19.5%	21	23.6%
pT stage	pT1	163	44.2%	24	29.7%
	pT2	171	46.3%	47	58.0%
	pT3	14	3.8%	6	7.4%
	pT4	21	5.7%	4	4.9%
pN stage	pN0	219	59%	39	45.7%
	pN1-2	150	41%	44	54.3%
Tumor grade	G1	39	10.6%	2	2.5%
	G2	162	43.9%	38	46.9%
	G3	168	45.5%	41	50.6%

Furthermore, we performed Kaplan-Meier analyses with respect to MFS in the overall collective as well as in the subset with normal *HER2* (82%) and amplified *HER2* (18%). Figure 1C illustrates the strong impact of *PLAUR* overexpression in the overall collective and the two *HER2* subsets. *PLAUR* overexpression correlated with poor outcome in the overall cohort of patients. Of interest *PLAUR* retained a significant impact also in the subsets with *HER2* amplification (See Figure 1C, *HER2* amplified). In the overall collective, the probability of MFS at 5 years for patients with *PLAUR* overexpressing tumors was 0.524 (CI: 0.373-0.735) as compared to 0.770 (CI: 0.718-0.826) for those with low *PLAUR* expression levels. These values decreased to 0.250 (CI: 0.075-0.830) and 0.716 (CI: 0.598-0.858) in the *HER2* amplified subset. Moreover, the Kaplan-Meier curves depicted better MFS for patients with *HER2* normal and low *PLAUR* phenotype tumors. In this case the following rates at five years were calculated: 0.601 (CI: 0.427-0.845) for high and 0.781 (CI: 0.723-0.843) for low *PLAUR* expression levels, respectively.

However, unlike the high and strong correlation between *HER2* and *PLAUR* mRNA expression, the correlation in the *HER2* and *PLAUR* gene co-amplification status in primary BC patients analyzed has been infrequent or absent. This is not surprising and is consistent with previous reports where *HER2* and *PLAUR* co-amplification status has been found to be a rare event across primary BC patients [68]. This is further evident in The Cancer Genome Atlas (TCGA) Breast Invasive Carcinoma Project data which involved analysis of primary BCs by genomic DNA copy number arrays, DNA methylation, exome sequencing, messenger RNA arrays, microRNA sequencing and reverse-phase protein arrays. This study found only one case of co-amplification of *HER2* and *PLAUR* in 825 primary BC patients [69, 70]. It should also be noted that by analyzing individual tumor cells the effect of averaging out gene amplification and/or expression status in tumors or their metastases is negated [33]. That is, significant associations between *HER2* and *PLAUR* gene co-amplification and co-expression may not be seen when biopsies of mixed cell populations are analyzed.

#### Common signaling molecules downstream of HER2 and uPAR

The hypothesis that high uPAR expression could be required for the invasive capacity of HER2 positive tumors was demonstrated by Tan et al., [34] who showed that uPA system contributes to a higher metastatic potential in HER2-overexpressing cancer cells. In HER2-overexpressing BC cells, Tan et al., [34] found upregulation and activation of protein kinase Cα (PKC*α*) through steroid receptor co-activator (Src) by HER2 to be critical for HER2-mediated cancer cell invasion. Other studies have found PKC*α* and Src to be critical components for uPAR-mediated cancer cell invasion in high uPAR expressing cancer cells [71, 72]. Tan et al. [34] also found that by inhibiting PKC*α* or Src by chemical inhibitors, dominant-negative mutants or siRNA, uPAR expression decreased and there was a reduction in cancer cell invasion in HER2 overexpressing BC cell lines. This indicates that HER2-mediated PKC*α*/Src upregulation and activation is required for the HER2-mediated upregulation of the uPAR, which may contribute to invasion and metastasis in HER2 positive tumors.

#### Src downstream of HER2 and uPAR

The p160 Src family contains 3 members: Src-1 (nuclear receptor co-activator 1 (NCOA1)), Src-2 (transcriptional intermediary factor-2 (TIF2), glucocorticoid receptor interacting protein-1 (GRIP1), or NCOA2), and Src-3 (amplified in BC-1 (AIB1), activator of retinoid and thyroid receptors (ACTR), or NCOA3) [73]. The SRC family members share an overall similarity of 50–55% in their amino acid sequences and interact with and coactivate other transcription factors such as ETS-2, PEA3, and E2F1 [74-83]. Numerous studies have been reported that show Src as a proto-oncoprotein of BC. Src binds to HER2 and is activated in HER2-overexpressing cancer cells [34, 84-86]. Among the Src family members, high Src-1 expression has been directly correlated with HER2 positivity, disease recurrence in HER2-positive BCs and resistance to endocrine therapy [77, 78], and disruption of the Src-1 gene in mice suppresses BC metastasis without affecting primary tumor formation [82]. Many other studies have also positively correlated Src with HER2 positive BC [87]. Meanwhile, Src has also already been shown to transduce signals from uPAR [88] providing mammary MCF-7 cells with a proliferative and invasive advantage.

#### HER2 and uPAR signaling mediated by PKCα

PKC family comprises of several isoforms that belongs to the family of serine/threonine kinases that regulate cell proliferation, differentiation, apoptosis, motility and adhesion [89, 90]. Various studies have found the PKC isoforms, mainly PKC*α,* PKCδ, and PKCε, to be highly expressed in BC cells imparting them with an increased invasive or metastatic potential than in normal tissues [34, 91-95]. For a detailed overview of the role of each of the PKC isoforms on tumorigenesis and BC in particular, refer to Lønne et al., [96]. Early experimentations by Peles et al., [97] showed HER2 to activate PKC*α* via phospholipase-*γ* (PLC *γ*). However, direct evidence on the exact regulatory role of PKC*α* expression in BC downstream of HER2 only came to known following studies by Tan et al., [34] as mentioned previously. Recently, Magnifico et al., [98] showed a specific physical association between PKC*α* and HER2 using solubilized lipid rafts and demonstrated PKC*α* mediated upregulation of HER2 expression and vice versa. They found, in HER2 positive BC cells, PKC*α* inhibition by pharmacologic treatments and PKC*α*-specific small interfering RNA (siRNA) led to a dramatic downregulation of HER2 levels. Consistent with this inhibition of HER2 activation by the tyrosine kinase inhibitor lapatinib led to decreased levels of PKC*α* phosphorylation. Thus PKC*α* has come to be recognized as a potential marker for BC aggressiveness. More importantly, Magnifico et al., [98] showed that HER2 overexpression in HER2 positive carcinomas is predominantly regulated by PKC*α* activity. The manifestation of this finding could be a regulatory loop where high PKC*α* expression maintains the HER2 overexpression and hence invasiveness. With respect to uPAR, studies reported as early as 1994 by Busso et al., [99] found that uPAR forms complexes with PKC in epithelial cells. Further to this, Sliva et al., [100] showed that inhibition of PKC represses constitutive (nonstimulated) migration of highly metastatic MDA-MB-231 cells with constitutively high levels of uPA. In this scenario, similar to PKC regulation of HER2 overexpression as mentioned earlier, it can be assumed that the constitutive uPA expression is maintained non-canonically by activation of PKC.

#### NF-κB pathway intermediates signaling from HER2 and uPAR

Both HER2 and uPAR are interlinked to NF-κB signaling. HER2 activates NF-κB signaling in HER2 overexpressing BC cell lines [101]. The canonical NF-κB family pathway, that is overexpressed in BC cells from both primary human tumors and in cell lines [102], mediates HER2-induced breast CSC expansion [103]. This finding implicating HER2 expression with CSC expansion is supported by several lines of evidence from independent studies. In one such study, the overexpression of HER2 correlated with the expression of the stem cell marker aldehyde dehydrogenase (ALDH) in BC patients [104]. Cicalese et al., [105] found that increased HER2 transgene expression in mice resulted in increased self-renewal and replicative potential for CSCs. In a separate *in vitro* study on BC cells, Korkaya et al., [106] found that HER2 overexpression increased the CSC population, as demonstrated by increased ALDH activity, mammosphere formation, tumorigenesis, and expression of stem cell related genes. On the other hand, NF-κB and other co-factors controlled the expression of uPA and uPAR, and the inhibition of NF-κB and activator protein-1 (AP-1) suppressed the secretion of uPA, resulting in the inhibition of motility of highly invasive BC cells [39, 100]. Therefore the role of NF-κB pathway downstream of both HER2 and uPAR assumes significance not only due to the part it plays in tumor initiation, metastasis and recurrence of disease condition with increased aggressiveness, but also from reports that implicate NF-κB to the expansion of CSCs. The latter finding implicating growth and metastasis of the tumor population in CSCs to be driven by HER2 and uPAR mediated by NF-κB may partially explain the failure of existing treatment strategies to completely eradicate solid tumors [107] and drug resistance. For example, one of the theories suggest that the efficacy of currently available drugs that can only shrink metastatic tumors are usually transient and does not lead to extended patient survival [108-110]. This has been blamed on the acquisition of drug resistance by the cancer cells and the failure to kill CSCs effectively by existing therapies. Therefore, the activation of non-canonical pathways through PKC and Src and canonical pathway mediated by NF-κB not only has implications in maintaining constitutive HER2 and uPAR overexpression and hence tumor invasiveness, but also could play a significant role in development of drug resistance.

**Figure 2:**
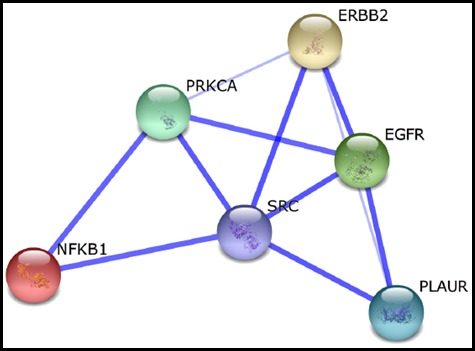
Protein functional interaction network for *ERBB2/HER2* and *PLAUR/uPAR* The proteins interacting with ERBB2 and uPAR were obtained from STITCH database 4.0. The nodes are formed by the individual proteins. The blue lines indicate the protein-protein functional interaction. Note that thicker lines indicate a stronger strength of functional interaction. Abbreviations: PLAUR, plasminogen activator, urokinase receptor; EGFR, epidermal growth factor receptor ;Src, v-src sarcoma (Schmidt-Ruppin A-2) viral oncogene homolog (avian); PRKCA, Protein kinase Cα; NF-κB (NFKB1), nuclear factor of kappa light polypeptide gene enhancer in B-cells 1).

As discussed before, both HER2 and uPAR appear to have functional interactions with downstream intracellular common oncogenic players such as Src, PKC*α*, and NF-κB. Hence, to further confirm these potential functional associations we used the interaction network database STITCH 4.0 [111] with multiple proteins option using input genes *PLAUR, ERBB2, PRKCA, NFKB1,* and *SRC*. All six input genes formed a single protein functional interaction network (Figure 2). This data analysis converges with previous findings implicating these molecules as critical factors in HER2 and uPAR-mediated invasion and metastasis of BC.

#### Common regulatory transcriptional factors of HER2 and PLAUR

**Figure 3:**
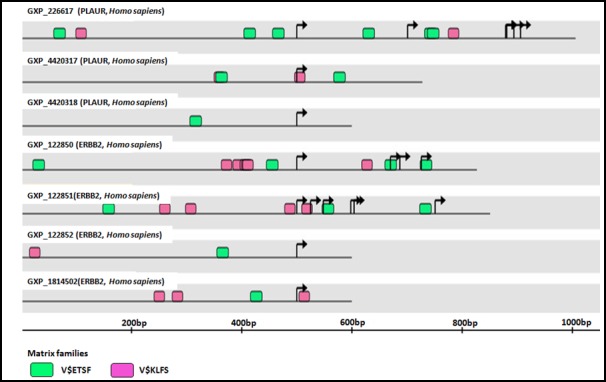
Schematic representation of common transcriptional factor binding sites (indicated by matrix family) for *ERBB2/HER2* and *PLAUR* Genomatix analysis identified alternative promoters for *PLAUR* and *ERBB2*. Note that the V$ETSF and V$KLFS family of transcription factors are common to both the promoters. Black arrows indicate the transcription start sites (TSS).

To identify the common transcription factors that have propensity to regulate both *HER2* and *PLAUR gene* expression in HER2-positive breast carcinoma, we submitted their gene symbols into GEMS launcher software. The analyzer identified V$ETSF (Ets family of transcription factors) and V$KLFS (Kruppel-like family of transcription factors) as the common transcription factor families (Figure 3) that bind to and regulate *HER2* and *PLAUR*.

### EGFR – A preferred dimerization partner of HER2 and an essential signal transducer for uPAR

Another important observation from the STITCH protein interaction network (Figure 2) is the strong association of EGFR with both HER2 and uPAR. Whilst several studies have shown HER2 to be the preferred dimerization partner of other HER family members [112], co-expression of HER2 with EGFR has been shown to induce a synergistic transforming effect on rodent fibroblasts [113]. A recent study has also found EGFR overexpression to be a poor prognostic factor in HER2-positive primary BC [114]. On the other hand, EGFR has also been demonstrated to mediate uPAR/integrin/fibronectin (FN) induced growth pathway leading to the *in vivo* proliferation of HEp3 human carcinoma [115]. This was further confirmed in a study by Jo et al. [116], where they found EGFR to be an essential component for the transduction of signals from uPAR to ERK in cells that express EGFR. More studies investigating the interactions between EGFR and uPAR followed. For example, Guerrero et al. [88] showed that in mammary epithelial MCF-7 cells expressing low levels of uPAR, stimulation of uPAR with the amino-terminal fragment (ATF) of urokinase devoid of proteolytic activity transactivated the EGFR through a mechanism involving Src and a metalloproteinase leading to cellular invasion. Monaghan-Benson et al. [117] found that binding of P25, a uPAR ligand, to uPAR causes an Src-dependent transactivation of EGFR and promotes the formation of EGFR-β1 complexes leading to upregulation of fibronectin matrix assembly. Jo et al. [118] further reported that uPAR is required for EGF-induced cell growth in MDA-MB 231 breast cancer cells and murine embryonic fibroblasts (MEFs) through Tyr-845 phosphorylation of EGFR and activation of STAT5b. D'Alessio et al. [119] found that mouse keratinocytes deficient for uPAR failed to produce and secrete EGFR-dependent laminin-5, affecting adhesion and migration properties *in vitro* and wound healing *in vivo*. Hu et al. [120] demonstrated uPAR to be a highly significant crosstalk molecule that is necessary for the activation of signal transducer and activator of transcription 5b (STAT5b), a recently identified downstream effector of EGFRvIII [121], in glioblastoma multiforme cells. A very recent study by Kozlova et al., [122] reported a uPA-uPAR mediated attenuation of the mitogenic effect of EGF on cellular proliferation, invasion and motility in MCF-7 and MDA-MB-231 breast cancer cells. Though interesting, more studies are needed to confirm in clinical specimens the role of uPA as a negative modulator of EGF-dependent cellular proliferation and motility.

### ETS members are transcriptional targets of HER2 and uPAR signaling

The ETS family of transcription factors are defined by a conserved DNA binding domain. This domain forms a winged helix-turn-helix structural motif [123]. Many ETS factors are shown to be dysregulated in BC such as ETS1 (v-ets avian erythroblastosis virus E26 oncogene homolog 1), ETS2 (v-ets avian erythroblastosis virus E26 oncogene homolog 2) and PEA3 (Polyomavirus enhancer activator 3) [124]. A handful of studies have addressed the ETS transcription factors mediated regulation of HER2 and uPAR signaling. ETS proteins have been implicated as downstream factors of HER2 signaling [125] and, at a clinical level, ETS proteins have been shown to associate with breast tumor disease progression and metastasis [74, 126]. These MAP kinase-dependent transcription factors interact with a multitude of co-regulatory partners to elicit a biological process [125, 127]. For example, studies by Myers et al., [75] found that Src-1 is a functional coactivator of ETS-2. Al-azawi et al., [74] reported strong associations between the transcription factor, ETS-2 and its coactivator Src-1 (*P*<0.01) and the target gene myc (*P*<0.0001) in a cohort of BC patients with locally advanced disease. On the other hand, PKC*α* was also found to regulate ETS1 activity as a downstream transcriptional factor in invasive BC cells [128]. On examining the PKC expression in a variety of BC cell lines, Lindermann et al., [128] found that the protein level of PKC*α* was much higher in ETS1-expressing MDA-MB-231 and MDA-MB-435 BC cells than in ETS1-deficient MCF-7 and SK-BR3 cells, whereas PKC*α*-deficient MCF-7 cells do not support ETS1-induced activation of the PTHrP P3 promoter strongly suggesting that PKC*α* may be important for ETS1 activity. To follow it up, studies also found that attenuation of endogenous PKC*α* expression (siPalpha) by RNA interference leads to reduced ETS1 protein expression in a variety of cancer cells suggesting that ETS1 serves as an effector for PKCα to fulfil certain functions in cancer cells [129].

### KLF transcription factors downstream of Src, PKCα, and NF-κB

KLFs are a diverse family of Zinc finger containing DNA binding transcription factors. Currently, 17 KLFs are known in mammals. They have a carboxy terminal DNA binding domain with three Zn fingers. Zn fingers bind to GC rich DNA sequences [130, 131]. Several KLFs are altered or elevated in cancer [131]. In BC, KLF2, KLF4, KLF5, KLF6, KLF8, KLF10 and KLF17 have been found to be altered [130, 132, 133]. For a detailed understanding KLF family members and its context dependent functions, refer to Tetreault et al., [133]. KLF4 expression is associated with BC progression and *KLF4* mRNA and protein are overexpressed in up to 70% of BCs [134, 135]. Increased nuclear expression of KLF4 is considered to be associated with the aggressiveness of BC phenotypes [135]. However, the precise role of KLF4 in transcriptional regulation of both the *PLAUR* and *HER2* in BC is yet to be examined in detail. Another KLF member, KLF8 promotes human BC cell invasion and metastasis by transcriptionally repressing cadherin 1 (*CDH1*) and transactivating matrix metallopeptidase (*MMP9*) [136, 137], and high expression of KLF8 predicts a poor prognosis in human cancers [133]. KLF10, on the other hand, transcriptionally represses *EGFR* and inhibits invasion and metastasis *in vitro* and in an orthotopic mouse tumor model [138], and KLF10 loss is downregulated in invasive human BC [133, 139]. Also, initial studies revealed KLF5 to be a potential tumor suppressor gene in BC [140], however, a recent study found that patients with a higher KLF5 expression have shorter disease-free and OS than patients with a lower KLF5 expression [132, 141]. It was recently shown that reduction or absence of KLF6 abrogates the negative control of BC cell proliferation triggered by ER–alpha through the signaling pathway mediated by c-Src and Akt activation [142]. In other words, cytoplasmic KLF6 is able to interact with c-Src protein and thereby interferes with ER–alpha-mediated cell growth of BC cells.

### HER2 and uPAR – Correlative markers and potential dual drug targets

**Figure 4:**
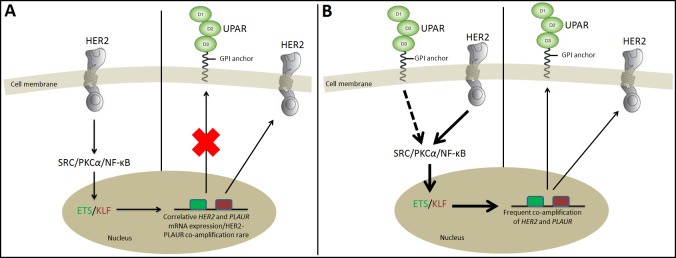
A model comparative diagrammatic representation of the regulatory signaling cascade in a primary/early metastatic HER2-positive breast carcinoma condition that also co-overexpress uPAR (A). In *HER2*-amplified primary BC, signals transduced from HER2 through SRC/PKC*α*/NF-κB leads to *HER2* and *PLAUR* mRNA co-expression, but no frequent *HER2* and *PLAUR* co-amplification has been observed. (B). However, in an early-stage aggressive HER2-positive BC condition, we propose that hyper-activation of HER2 transduces strong signals (bold arrows) through SRC or PKC*α* or NF-κB individually or in a concerted manner, leading to activation of members of ETS or KLF family. Consequently, binding of ETS or KLF family members on the promoter region of *HER2* or *PLAUR* gene, leads to their co-amplification, thereby facilitating the high expression of uPAR and HER2 in HER2-positive BC subtype. Depending on the downstream effectors (SRC or PKC*α* or NF-κB) mediating the signaling pathway, one or more members of the ETS and KLF family will be involved in the regulation of *HER2* and *PLAUR* gene amplification. According to literature, high expression of uPAR is associated with invasive potential of BC. Therefore, it can be assumed that high uPAR expression gives the invasive advantage to the early stage aggressive HER2-positive BC condition, which is reflected in the high metastatic potential of most of the HER2-positive BC subtype that co-overexpress uPAR. Also, depending on the availability and binding of endogenous uPA to uPAR, the signaling cascade initiated from uPAR in association with integrin family of receptors or GPCRs can also increase the expression of HER2 and uPAR at the cell surface following signaling mediated by SRC/PKC*α*/NF-κB (represented by dotted arrow), leading to the activation of ETS and KLF transcriptional factors that regulate *HER2* and *PLAUR* gene amplification. Green box represents binding site of ETS on HER2 or uPAR promoter region. Red box represents binding site of KLF on *HER2* or *PLAUR* promoter region.

It has been proposed that amplification of a single chromosomal region (for example, *HER2*) may destabilize the tumor genome, thereby facilitating the amplification of an additional loci [68, 143] (for example, *PLAUR*). If these amplification combinations were to exist, breast tumors harbouring *HER2* gene amplification can be assumed to acquire subsequent amplification of the *PLAUR* gene at a later stage of tumor development, thereby allowing the tumor cells to acquire the ability to invade the surrounding tissues and spread to distant sites of the body [68]. Regulation of *PLAUR* and *HER2* in advanced BCs by common oncogenic players as evident from the preclinical evidence, STITCH database analysis (Figure 2), and common transcriptional factor binding sites (Figure 3) confirms the role of Src, PKC*α*, and NF-κB signaling downstream of HER2 and uPAR in altering the amplification status of *PLAUR* and *HER2*. Therefore, in HER2-positive early-stage aggressive breast carcinoma, it could be likely that the hyper-activation of common oncogenic players like Src, PKCα and NF-κB may act in a non-canonical mechanism either independently or in a concerted manner to upregulate the amplification status of *PLAUR* and/or *HER2* through activation of common transcription factors such as ETS and KLF (Figure 4A & B). This partly explains the existence of *PLAUR* amplification in CTCs from patients with HER2-positive BC [33] and is consistent with previous reports that showed marked preference for amplification of both *HER2* and *PLAUR* genes to occur in the same CTC in HER2-amplified tumors [144, 145]. Since CTCs have been reported recently as precursors and contribute significantly to BC metastasis [146], high *HER2* and *PLAUR* co-amplification can be expected to be seen only during the later stages of malignant tumor development (CTCs to distant metastases stage). Therefore, upregulation of *HER2* and *PLAUR* by common oncogenic players can be attributed as being specific to an early-stage aggressive breast carcinoma subtype.

According to the Tumor Marker Utility Grading System, uPA and PAI-1 invasion markers along with HER2 are still the most dominant independent novel prognostic factors that have reached the highest level of evidence for clinical utility in BC [4, 147, 148]. It is evident from previous studies [149, 150] and Figure 1B, that uPA is highly correlated with uPAR in BC. Various studies have shown uPA/PAI-1 and HER2 as independent prognostic and predictive markers for DFS and on aggressive outcome in lymph node-negative BC [6, 151-154]. At the same time, uPA and PAI-1 mRNA expression have been shown to have a strong association with shorter DFS (p = 0.013 for PAI-1, p = 0.001 for uPA) in HER2-positive BC patients [35, 155]. Currently, the main clinical relevance of uPA/PAI-1 as prognostic biomarkers is in the identification of lymph node-negative patients with HER-2-negative tumors for adjuvant chemotherapy [152]. However, the combined clinical relevance of HER2 and uPA/PAI-1 can significantly contribute towards optimal decision making in the selection of patients with primary BC for various treatment strategies. Since uPA and uPAR strongly correlate (Figure 1B), and patients with tumors expressing high uPA, high uPAR, and/or high PAI-1 levels show a significantly shorter RFS and OS compared to patients with low levels of their expression [150], the assessment of both markers together with HER2 and PAI-1 in BCs will enable clinicians to accurately predict the disease outcome and to identify in early stage patients, who will benefit from combined therapies.

In the light of the HER2 and uPAR cooperativity and the common regulatory signaling pathway downstream of HER2 and uPAR in advanced breast carcinoma (Figure 4B), the correlative co-expression pattern of HER2 and uPAR definitely has the potential to act as synergistic targets for therapeutic intervention. This suggestion is well supported by studies done by Li et al., [157] who found that downregulation of HER2/uPAR individually at the cell surface, leads to decreased ERK activity and this effect maximizes upon downregulation of both receptors simultaneously indicating a synergistic effect on BC cells. Li et al., [157] further demonstrated that RNA interference (RNAi) depletion of either HER2 or uPAR suppressed cell growth and induced cell apoptosis, and these effects were significantly enhanced in cells depleted of both HER2 and uPAR. Moreover, downregulation of uPAR using RNAi synergized with trastuzumab to suppress the growth and induce apoptosis of SKBR3 and ZR751 cells and this effect was also evident in the mechanistic analysis where uPAR RNAi significantly enhanced the effect of trastuzumab on inhibition of MAPK signal pathways. This recent finding makes these receptors potential targets for combinatorial therapies using either trastuzumab and uPAR antagonists or selective small molecules or antibody-drug conjugates to achieve inhibition of HER2 and uPAR. It can be expected that simultaneous targeting of HER2 and uPAR, the cooperativity of which this review discusses and substantiates to contribute to the metastatic phenotype of HER2-positive BC, may possibly convert a cell's phenotype from tumorigenic to dormant or prolong their dormant state with less adverse side effects.

## CONCLUSION

Previously, the model of metastasis was explained in terms of rare subpopulations of cells within the primary tumor that acquire advantageous genetic alterations over a period of time, enabling these cells to metastasize and form new solid tumors at distant sites [158]. This genetic selection model of metastasis was debated for some time by various groups [159-161], before the emergence of gene expression profiling data [162-164]. Studies based on DNA microarrays reported that primary breast tumors can be distinguished by their gene expression profile for their metastatic potential. This implies that genetic mutations determine metastatic behavior at early stages of tumorigenesis [165]. This review confirms previous knowledge and substantiates non-canonical mechanisms contributing to the cooperativity between HER2 and uPAR in advanced BC. This process involves various other downstream molecules including Src/PKC*α*/NF-κB leading to the activation of transcriptional factors such as ETS or KLF that contribute to the aggressiveness of HER2-positive breast carcinoma phenotype and possibly cause feed-back resistance mechanism to HER2 targeted therapy. Based on the critical nature of cooperativity between HER2 and uPAR in advanced HER2-positive breast carcinoma, this review also stresses the importance of targeting simultaneously HER2 and uPAR to improve personalized treatment modalities of newly diagnosed patients.

## Abbreviations

BC: Breast cancer
FDA: Food and Drug Administration
uPAR: urokinase plasminogen activator receptor
uPA: urokinase plasminogen activator
PAI-1: plasminogen activator inhibitor type 1
RFS: Relapse-free survival
HER2: Human epidermal growth factor receptor type 2
DFS: disease-free survival
CTCs: circulating tumor cells
DTCs: disseminated tumor cells
ER: estrogen receptor
PR: progesterone receptor
NF-κB: nuclear factor-kappaB
CSCs: cancer stem cells
GPI: glycosyl phosphatidylinositol
GPCR: G-protein-coupled receptor
Lck: lymphocyte protein tyrosine kinase
Hck: haematopoietic cell kinase
FAK: focal adhesion kinase
ERK: extracellular-signal-regulated kinase
MAPK: mitogen-activated protein kinase
MFS: metastases-free survival
HDPP: HER2-derived prognostic predictor
OS: overall survival
DMFS: distant metastasis-free survival
NKI: Nederlands Kanker Instituut
FFPE: formalin-fixed and paraffin-embedded
RPPA: reverse phase protein microarray
*PKCα*: Protein kinase Cα
Src: steroid receptor co-activator
NCOA1/NCOA2/NCOA2: nuclear receptor co-activator 1/2/3
TIF2: transcriptional intermediary factor-2
GRIP1: glucocorticoid receptor interacting protein-1
AIB1: amplified in BC-1
ACTR: activator of retinoid and thyroid receptors
PLC*γ*: phospholipase-*γ*
siRNA: small interfering RNA
ALDH: aldehyde dehydrogenase
AP-1: activator protein-1
EGFR: epidermal growth factor receptor
FN: fibronectin
ATF: amino-terminal fragment
STAT5b: signal transducer and activator of transcription 5b
ETSF: Ets family of transcription factors
KLFS: Kruppel-like family of transcription factors
PEA3: Polyomavirus enhancer activator 3
*CDH1*: cadherin 1
*MMP9*: Matrix metallopeptidase
TCGA: The Cancer Genome Atlas
RNAi: RNA interference.
